# The effects of ginseng on the metabolic syndrome: An updated review

**DOI:** 10.1002/fsn3.2475

**Published:** 2021-07-20

**Authors:** Tahereh Aminifard, Bibi Marjan Razavi, Hossein Hosseinzadeh

**Affiliations:** ^1^ Department of Pharmacodynamics and Toxicology School of Pharmacy Mashhad University of Medical Sciences Mashhad Iran; ^2^ Targeted Drug Delivery Research Center Pharmaceutical Technology Institute Mashhad University of Medical Sciences Mashhad Iran; ^3^ Pharmaceutical Research Center Pharmaceutical Technology Institute Mashhad University of Medical Sciences Mashhad Iran

**Keywords:** diabetes, ginseng, herbal medicine, hypertension, metabolic syndrome, *Panax ginseng*

## Abstract

Metabolic syndrome is a group of risk factors including high blood glucose, dyslipidemia, high blood pressure, and high body weight. It can increase the risk of diabetes and cardiovascular disorders, which are the important reasons for death around the world. Nowadays, there are numerous demands for herbal medicine because of less harmful effects and more useful effects in comparison with chemical options. Ginseng is one of the most famous herbs used as a drug for a variety of disorders in humans. The antihyperlipidemia, antihypertension, antihyperglycemic, and anti‐obesity effects of ginseng and its active constituents such as ginsenosides have been shown in different studies. In this review article, the different in vitro, in vivo, and human studies concerning the effects of ginseng and its active constituents in metabolic syndrome have been summarized. According to these studies, ginseng can control metabolic syndrome and related diseases.

## Graphical abstract

Metabolic syndrome is a group of risk factors including high blood glucose, dyslipidemia, high blood pressure, and high body weight. Ginseng is one of the most famous herbs used as a drug for a variety of disorders in humans. According to the different in vitro, in vivo, and human studies, ginseng can control metabolic syndrome and related diseases.

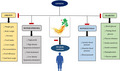

## 1 INTRODUCTION

Metabolic syndrome or syndrome X is the name given to the collection of clinical conditions including overweight, high blood pressure, high blood glucose (or type 2 diabetes mellitus), and hyperlipidemia (Chen et al., 2017).

Metabolic syndrome is the most important risk factor for atherosclerosis in response to chronic inflammation and vascular endothelial dysfunction. Metabolic syndrome increases the risk of cardiovascular diseases. Cardiovascular diseases are the main reasons for mortality around the world (Kang & Park, 2012). The metabolic syndrome is rising due to a sedentary lifestyle. Children, adolescents, and young women with polycystic ovary syndrome are at risk for metabolic syndrome (Vassallo et al., 2016).

Medicine herbs, because of their potential efficacy in improving and holding human health, low cost, and adverse effects, have been the focus of attention. Studies have been shown that several plants and their active constituents can exert beneficial effects on metabolic syndrome. For example, grapes *(Vitis vinifera*), a source of polyphenol antioxidants, is useful for preventing the risk factors involved in metabolic syndromes such as hyperlipidemia, hypertension, and hyperglycemia (Akaberi & Hosseinzadeh, 2016). Garlic (*Allium sativum*) has been documented in the treatment of metabolic syndrome as it showed hypoglycemic, hypotensive, and hypolipidemic activities (Hosseini & Hosseinzadeh, 2015). Rosemary *(Rosmarinus officinalis* L.) is a source of phenolic phytochemicals having considerable anti‐inflammatory, antioxidant, hypoglycemic, hypolipidemic, and hypotensive effects (Hassani et al., 2016).

*Panax ginseng* belonging to the genus *Panax* and the family Araliaceae is one of the popular pharmaceutical and perennial plant species. The plant is cultivating in China, Japan, and Korea (Lee et al., 2017).

Its curative function for the first time was seen in the Chinese medicine monograph (Yun, 2001). Ginseng is one of the most famous herbs used as a drug and nutritional supplements for a variety of disorders in humans (Liu et al., 2019b).

“*Panax*” taken from the word “panacea” in Greek which means “cure‐all.” Ginseng has been proven to have a wide variety of therapeutic effects. Red ginseng and white ginseng are two prevalent products of ginseng. Red ginseng provided during the process of steaming, and dried white ginseng provided by air‐drying (Karmazyn et al., 2011). Red ginseng has shown efficacy for the remedy of a wide range of disorders including hyperglycemia (Nam et al., 2019).

**FIGURE 1:**
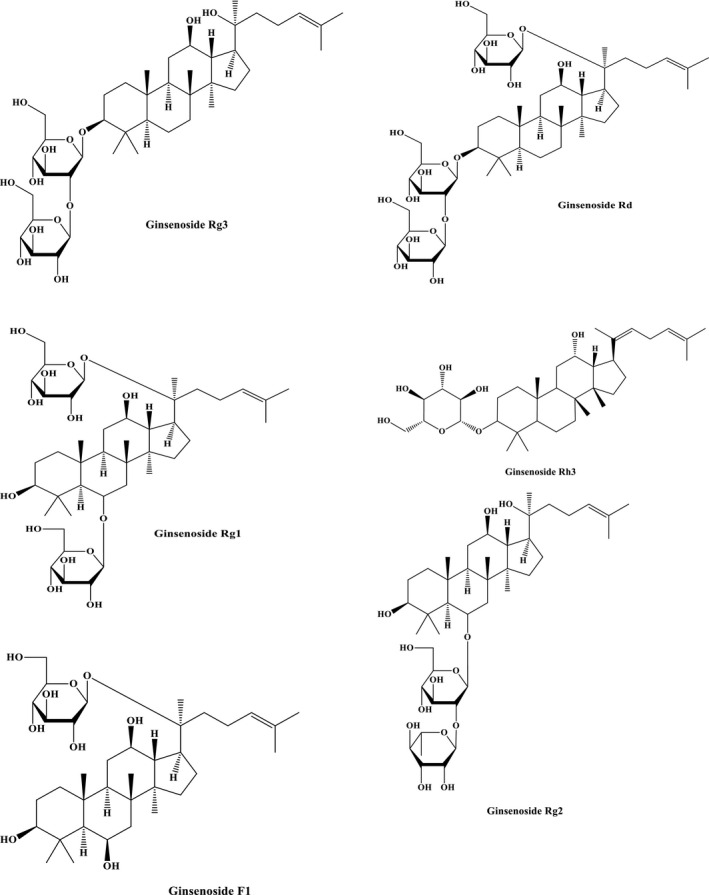
Some of the pharmacologically active constituents of ginseng

The important bioactive structures in ginseng are ginsenosides, the different types of triterpene saponins including oleanane‐type ones and dammarane‐type ones, that classified according to their chemical skeleton structures (Han et al., 2006). Until now, over than 150 ginsenosides have been purified from ginseng; in *Panax ginseng*, 40 kinds of ginsenosides have been found (Christensen, 2008). Some of the most active constituents of ginseng are structurally shown in Figure 1. Ginsenosides are the principal group of effective compounds in ginseng. They demonstrate unique biological activity and broad pharmacological properties including anticancer, anti‐inflammatory, antioxidant, and anti‐apoptotic effects (Razgonova et al., 2019).

According to the modern pharmacological, preclinical, and clinical studies, ginseng has marvelous beneficial effects in multiple neurological and neurodegenerative diseases and it has various biological activities such as antihypertension, antitumor, anti‐anxiety, and immune‐modulatory activities, so it has several protective mechanisms (Liu et al., 2019b). Furthermore, ginseng has wide curative potentials including diminishing blood glucose, modifying blood lipids, and enhancing insulin sensitivity (Imanshahidi & Hosseinzadeh, 2008). Therefore, ginseng is effective in the treatment of different components of metabolic syndrome.

In this review, different relevant studies to realize the role of ginseng and its active components in metabolic syndrome risk factors including hypertension, hyperglycemia, hyperlipidemia, and obesity have been explained.

## 2 METHODOLOGY

This review was carried out by the means of the databases of Scopus, PubMed, and Web of Science. All the articles in this review were collected from 2009 to 2019. The search keywords contain “metabolic syndrome”, hypertension, “blood pressure”, hypotensive, antihypertensive, hypertensive, diabetes, hyperglycemia, insulin, hypoglycemic, antihyperglycemic, antidiabetic, “blood glucose”, dyslipidemia, hyperlipidemia, "high cholesterol", "high triglyceride", hypercholesterolemia, hypertriglyceridemia,atherogenic, atherosclerosis, obesity, overweight, appetite, anti‐obesity, “weight loss”, “bodyweight”, “food intake”, “feed intake”, ginseng, ginsenoside, and panaxsoide.

## 3 EFFECT ON DYSLIPIDEMIA

Hyperlipidemia is found as an important risk factor for heart and vessel disorders, which is one of the important reasons for human mortality (Mueller et al., 2011).

Recently, there has been an enhancing interest in the usage of medicinal herbs with more efficiency and lower adverse effects than chemical drugs for a variety of disorders including hyperlipidemia.

According to the studies focused on the plants with hypolipidemic effects, some plants including *Allium sativum*, *Nigella sativa*, *Curcuma longa*, *Anethum graveolens*, and *Commiphora mukul* showed the best hypolipidemic effect (Mollazadeh et al., 2019).

Numerous studies have been shown that ginseng could decline total cholesterol (TC), triglyceride (TG), and low‐density lipoproteins (LDL) and increase high‐density lipoprotein (HDL) level.

A study on male and female rats intoxicated with ethanol for two weeks indicated that the administration of 150 mg/kg of ginseng for six weeks improved the serum lipid profiles. Ginseng lowered the serum level of TC, TG, LDL‐C, and atherogenic index and elevated the serum level HDL‐C (Ayaz & Alnahdi, 2018). In another study in mice receiving the alcoholic extract of North American ginseng at 4 and 32 weeks of age, a decrease in hepatic and intestinal lipoprotein secretion and the level of blood lipid has been shown. Treatment by ginseng protected the mice against fatty liver. Moreover, ginseng reduced the expression of genes involved in the adjustment of fatty acid and triglyceride secretion by the lipoproteins. On the other hand, ginseng stimulated lipolysis (Singh et al., 2017). The protective effects of ginseng on dyslipidemia may be related to the increased phosphorylation of AMP‐activated protein kinase (AMPK) and acetyl‐CoA carboxylase. Also, the different gene expressions related to lipolysis and uptake of fatty acids such as peroxisome proliferator‐activated receptor‐α and CD36 were enhanced. These results have shown that ginseng improved hyperlipidemia by stimulating lipolysis through AMPK activation (Yuan et al., 2010). Administration of fermented red ginseng for eight weeks could reduce the levels of ALT, AST, TC, TG, and LDL‐C and hepatic MDA levels in high‐fat diet‐treated rats. Moreover, ginseng improved the HDL‐C level and hepatic SOD, CAT, and GSH‐Px activity induced by a high‐fat diet. These results demonstrated that ginseng modified lipid profiles, prevented lipid peroxidation, and increased antioxidant activities (Kim, Lee, et al., 2016). Also, Saba *et al*. showed that the administration of aqueous and ethanolic extracts of ginseng decreased the cholesterol and LDL levels in HFD‐treated rats. It also downregulated the important genes responsible for lipogenesis, such as acetyl‐coenzyme A (CoA) acetyltransferase 2, 3‐hydroxy‐3‐methyl‐glutaryl‐CoA reductase, and sterol regulatory element‐binding protein 2 (Saba et al., 2016). According to these studies, it could be concluded that ginseng could modify the lipid profile and inhibit atherosclerosis (Table 1).

**TABLE 1:** Effects of ginseng on lipid profile

Study design	Constituents	Result	References
In vivo, male and female rats (intoxicated with ethanol for two weeks)	150 mg kg^−1^ day^−1^ Ginseng for 6 weeks/P.O	↓ TG, ↓ TC, ↓ LDL‐C, ↑ HDL‐C	Ayaz and Alnahdi, (2018)
In vivo, Wistar rats, Fed HFD	0.2 g kg^−1^ day^−1^ of Ginseng for 90 days/ P.O	↓ TC	Akdoğan et al., (2018)
In vivo, ETKO obese mouse	200 mg kg^−1^ day^−1^ NAGEE, for 4 weeks / P.O	↓ HILS, ↓ CLL	Singh et al., (2017)
In vivo, Sprague Dawley rats, Fed HFD	200 mg kg^−1^ day^−1^ GWEE, for 4 weeks/ P.O	↓ TC,↓ LDL‐C	Saba et al., (2016)
In vivo, Rats, Fed HFD	Cotreatment with FBR and GE 250 mg kg^−1^ day^−1^, for 12 weeks/ P.O	↓ TG, ↓ TC, ↓ LDL‐C, ↑ HDL‐C, ↑ FEC	Lee et al., 2015)
In vivo, Male white rabbits, Fed HFD	RGNK and RG 0.11%(w/w) and NT 0.11%(w/w), P.O / for 8 weeks	RG: ↓ TG RGNK : ↓ TG, ↓ HC, ↓CETPAL	Kang et al., (2014)
In vivo, C57BL/6J mice, Fed HCD	Cotreatment with UBR and RG, 125 mg kg^−1^ day^−1^ for 12 weeks	↓ TC, ↓ HTL, ↓ LA	Lee, Lee, et al., (2014)
In vivo, ICR mice, fed HFD	PPGR, 75, 150, 300 mg/Kg/day, P.O, for 5 weeks	↓ Hyperlipidemia	Yuan et al., (2012)
In vivo, C57BL/6J mice, Fed HFD	GLE, 250 and 500 mg kg^−1^ day^−1^, P.O, for 8 weeks	↓ TG, ↓ TC, ↓ NEFA, ↓ LLD	Yuan et al., (2010)
In vivo, Sprague Dawley rats, Fed HFD	Ginseng 0.5% and 1%, daily, P.O, for 8 weeks	↓ TG, ↓ TC, ↓ LDL ‐C, ↑ HDL‐C, ↓ Lipid peroxidation	Kim, Lee, et al., (2016)
In vivo, Rat, Fed HFD	Cotreatment 2 ml/kg *Nigella sativa* oil and 6ml/kg Ginseng, daily, P.O, for two weeks	↓ BL, ↓Harmful changes in lipids	Arab et al., (2016)
In vivo, STZ‐induced diabetic rats	FRG, 100 and 200 mg kg^−1^ day^−1^, P.O, for 3 weeks	↓ TG, ↓ TC	Kim et al., (2010)
In vivo, STZ‐induced diabetic rats	PG, 22.5 mg kg^−1^ day^−1^, P.O, for 45 days	↓ TG, ↓ TC, ↓ LDL‐C, ↓ VLDL Cholesterol	El‐Khayat et al., (2011)
In vivo, STZ‐induced diabetic rats	PFRG, 300 mg kg^−1^ day^−1^, P.O, for 5 weeks	↓ TC	Park et al., (2012)
In vivo, Type 2 diabetic mice (db/db mice)	MEARG, 150 mg kg^−1^ day^−1^, IP	↓ TC, ↓ LDL‐C, ↑ HDL‐C	Yoo et al., (2012)
In vivo, HFD and STZ‐induced type 2 diabetic rat	MGR from PG, 50 and 100 mg kg^−1^ day^−1^, for 3 weeks	↓ TG, ↓ TC	Liu et al., (2013)
In vivo, Albino rat, Suffering from acute liver diseases and diabetes	Ginseng (2% and 4%) and cotreatment (2% Ginseng and 0.25% Curcumin) and (4% Ginseng and 0. 5% Curcumin), PO	↓ TG, ↓ TC, ↓ LDL‐C, ↓ VLDL Cholesterol, ↑ HDL	Arafa (2013)
In vivo, C57BL/6N mice, Fed HFD	PG Meyer, 4.0%, PO, for 8 weeks	↑ Lipid metabolism	Im Chung et al., (2016)
In vivo, Type 2 diabetic rat	*Baihu ginseng* decoction, 37.2 g kg^−1^ day^−1^, PO, for 2 weeks	↓ TG, ↓ TC, ↑ HDL‐C	Hou et al., (2017)
In vivo, STZ‐induced diabetic mice	RGPF with probiotics, 150 mg kg^−1^ day^−1^, PO, for 8 weeks	Improved serum lipid levels	Jang et al., (2017)
In vivo, C57BL/6 Mice, Fed HFD	Ginsenoside Re, 5, 10, and 20 mg kg^−1^ day^−1^, for 4 weeks	↓ TC, ↓ LDL‐C, ↑ HDL‐C, ↓ TG	Kim et al., (2017a)
In vivo, STZ‐induced type 2 diabetic C57BL/6 mice, Fed HFD	Ginsenoside Rk3, 10, 30, 60 mg kg^−1^ day^−1^, for 4 weeks	↓ TG, ↓ TC, ↓ LDL ‐C	Liu et al., (2019c)
In vivo, High‐fat and high‐sugar induced HIR in rat	Ginsenoside Rg1, 10, 25, 50 mg kg^−1^ day^−1^, PO, for 4 weeks	↓ TC, ↓ LDL‐C, ↑ HDL‐C, ↓ TG	Fan, Zhang, et al., (2019)
In vivo, Obese mice, fed HFD	Ginsenoside Rg2, 10 mg kg^−1^ day^−1^, PO, for 4 weeks	↓ FFA ↓ TG	Liu et al., (2019b)
In vivo, Obese mice, Fed HFD	GE, 0.8 and 1.6 (w/w) daily, PO, for 8 weeks	↓ TG	Lee et al., (2010)
In vivo, Mice, Fed HFD	Ginsenoside Rh2, 20 mg kg^−1^ day^−1^, PO, for 4 weeks	↓ TG	Gu et al., (2013)
In vivo, C57BL/6 mice, Fed HFD	Saponins from PG, 125 and 500 mg kg^−1^ day^−1^ for 12 weeks	↓ TG, ↓ TC, ↓ LDL ‐C, ↓ FFA	Chen et al., (2017)
In vivo, C57BL/6J mice, Fed HFD	Ginseng‐plus‐*Bai‐Hu‐Tang* 0.5% (w/w), PO, for 12 weeks	Protected against hyperlipidemia	Lu et al., (2018)
In vivo Castrated C57BL/6J mice, Fed HFD	GE, 5% for 8 weeks	↓ TG, ↓ TC, ↓ HLA	Shin and Yoon (2018)
In vivo, Obese C57BL/6 mice, Fed HFD	FGR and FGB for 16 weeks	↓ Hypercholesterolemia and fatty liver	Li et al., (2018)
In vivo, Mice, Fed HFD	Ginsenoside, 0.02% and 0.05% (w/w), for 8 weeks	↓ Hypertriglyceridemia	Liu et al., (2010)
In vivo, Mice, Fed HFD	HGV, 0.8 ml kg^−1^ day^−1^ for 8 week	↓ TG, ↓ LDL‐C, ↑ HDL‐C	Oh et al., (2014)
In vivo, Mice, Fed HFD	Cotreatment FG 150 mg kg^−1^ day^−1^ and 100 mg kg^−1^ day^−1^ of *Levan* for 11 weeks	↓ TC	Oh, Lee, et al., (2014)
In vitro, 3T3‐L1 adipocytes, Cultured under high cholesterol or fatty acids conditions	Ginsenoside Rb2, 10 μg/ml	↓TC, ↓ TG	Kim et al., (2009a)
Human, type 2 diabetic subject	Cotreatment KGB 6 g and AG 3 g, for 12 weeks	↓ Lipid concentration	Yang et al., (2018)
Human, 45 subjects (men and women)	RG, 20 g/day for 8 weeks	Improved lipid profile	Shin et al., (2011)

Abbreviations: AG, American ginseng; BL, blood lipids; CETPAL, cholesteryl ester transfer protein activity levels; CLL, circulating lipids level; FBR, fermented black raspberry; FCE, fecal cholesterol excretion; FFA, free fatty acid; FG, fermented ginseng; FGB, fermented ginseng berry; FGR, fermented ginseng root; FRG, fermented red ginseng; GE, ginseng extract; GE, ginseng extracts; GLE, ginseng leaf extract; GWEE, ginseng water and ethanol extract; HC, hepatic cholesterol; HCD, high cholesterol diet; HDL‐C, high‐density lipoprotein cholesterol; HFD, high‐fat diet; HGV, hydroponic‐cultured ginseng vinegar; HILS, hepatic and intestinal lipoprotein secretion; HIR, hepatic insulin resistance; HLA, hepatic lipid accumulation; HTL, hepatic total lipid; IP, intraperitoneal; KGB, *Konjac‐glucomannan*‐based fiber blend; LA, lipid accumulation; LDL‐C, low‐density lipoprotein cholesterol; LLD, liver lipid droplets; MEARG, methanolic extract of American Red Ginseng; MGR, malonyl ginsenosides; NAGEE, North American ginseng ethanol extract; NEFA, nonesterified fatty acids; NT, nattokinase; PFRG, puffed and fermented red ginseng; PG, *Panax ginseng*; PPGR, pectinase‐processed ginseng radix; RG, red ginseng; RG, red ginseng; RGE, red ginseng extracts; RGNK, RG combined with NT; RGPF, red ginseng powder fermented; STZ, streptozotocin; TC, total cholesterol; TG, triglyceride; UBR, unirpe black raspberry; VLDL, very‐low‐density lipoprotein.

## 4 EFFECT ON HIGH BLOOD PRESSURE

Hypertension is an additional important metabolic risk factor for cardiovascular disease (Organization, 2007). High blood pressure increases the risk of myocardial infarction and cerebrovascular disease as well as heart failure, peripheral vascular disease, stroke, and coronary artery disease (Leong et al., 2013).

Medicinal herbs are accessible, cheap, and useful for both prevention and treatment of hypertension. Furthermore, some medicinal plants such as *Ginkgo biloba* (Eisvand et al., 2020), *Aloe vera* (Shakib et al., 2019), *Crataegus pinnatifida* (Dehghani et al., 2019), *Silybum marianum* (Tajmohammadi et al., 2018), *Capsicum annuum* (Sanati et al., 2018), *Berberis vulgaris* (Tab eshpour et al., 2017), *Persea americana* (Tab eshpour et al., 2017), *Cinnamomum verum* (Mollazadeh & Hosseinzadeh, 2016), *Crocus sativus* L. (saffron) (B. M. Razavi & Hosseinzadeh, 2017), and *Nigella sativa* (B. Razavi & Hosseinzadeh, 2014) appear to have a great antihypertensive effect.

According to animal and human studies, ginseng can reduce hypertension. In a study, the administration of ginseng to spontaneously hypertensive rats improved endothelium‐dependent vasodilatation. Ginseng treatment for 6 weeks increased the serum NO levels and decreased the mean aortal intima–media width in comparison with control. Furthermore, ginseng mediated the expressions of cyclooxygenase (COX)‐2 in endothelial cells (Park et al., 2014b). Clinical investigations also demonstrated that the daily consumption of capsules of ginseng is helpful for blood flow during exercise. Ginseng reduced peripheral vascular resistance and improved oxygen transfer to activate atrophied muscles (Zaheri & Marandi, 2016). In another animal study, concurrent treatment with ginseng reduced the blood pressure in spontaneously hypertension rat by the inhibition of angiotensin‐I‐converting enzyme and release of NO (Lee, Bae, Park, Park, Lee, 2016). A study on prehypertensive subjects revealed that treatment by ginseng decreased lipoprotein‐associated phospholipase A 2 (Lp‐PLA 2) and lysophosphatidylcholines (lysoPCs) and increased dihydrobiopterin levels, which caused a notable decrease in diastolic and systolic blood pressure (Cha et al., 2016). Besides, the oral administration of ginseng to healthy volunteers for two separate appointments with a 7‐day washout course demonstrated that ginseng extract reduced central and peripheral arterial pressures in healthy adults (Jovanovski et al., 2014). A study on hypertensive diabetic patients showed that 3 g of ginseng for 12 weeks improved arterial stiffness and decreased the systolic blood pressure (Mucalo et al., 2013). Ginseng also showed hypotensive effects in combination therapy in rats, for example, a combination including *P*. *ginseng*, *P*. *notoginseng* (Burk.), and *Ligusticum chuanxiong* reduced the expression of cytokines leading to reduce aging and hypertension (Lei et al., 2012). According to the results of a study on hypotension induced by ginseng in spontaneously hypertensive rats, it can be suggested that ginseng is useful in decreasing high blood pressure through eNOS activation and enhanced NO‐releasing (Hong et al., 2012).

Several in vivo and clinical studies reported the antihypertension effect of ginseng and its protection against hypertensive complications such as cardiac hypertrophy (Table 2).

**TABLE 2:** Effects of ginseng on high blood pressure

Study design	Constituents	Result	Reference
In vivo, Spontaneously hypertensive rat	EEG, 50% and 85%, daily, for 6 weeks	↓ Mean aortal intima–media width	Park et al., (2014b)
In vivo, Spontaneously hypertensive Wistar Kyoto rat	Ginseng, 500 and 1,000 mg kg^−1^ day^−1^ for 8 weeks	↓ BP	Lee, Bae, et al., (2016)
In vivo, Mice, Fed HFD	Ginseng, 0.5 g kg^−1^ day^−1^, for 15 weeks	↓ BP	Li et al., (2014)
Human, Prehypertensive subjects	Ginseng, 5 g, daily for 12 weeks	↓SBP ↓DBP	Cha et al., (2016)
Human, Athletes men	Ginseng, 2 capsules−200 mg kg^−1^ day^−1^, for 4 weeks	Helpful for blood flow during exercise, ↓ Peripheral vascular resistance, improved oxygen transfer to muscles	Zaheri and Marandi (2016)
Human, Healthy subjects (male and female)	Ginseng, 400 mg on two separated appointments with a 7‐day washout course	↓ Central and brachial arterial pressure, ↓Arterial pressure ↓ SBP ↓ DBP	Jovanovski et al., (2014)
Human, Healthy subjects	Ginseng, 100 mg and 300 mg with 2 weeks washout period	↓ BP after 4 weeks, especially at high dose. Over 8 weeks, the effect is not retained	Rhee et al., (2014)
Human, Diabetic patients	Ginseng, 3 g/day for 12 weeks	Improved arterial stiffness and weakened SBP	Mucalo et al., (2013)
Human, Hypertensive subject	Ginseng, 3 g/day for 3 months	Ginseng did not progress arterial stiffness in hypertension patients.	Rhee et al., (2011)
Human, Type 2 diabetic subject	AG, 3 g/day, for 8 weeks	↓SBP	Vuksan et al., (2019)

Abbreviations: AAG, American ginseng; BP, blood pressure; DBP, diastolic blood pressure; EEG, ethanolic extract ginseng; HFD, high‐fat diet; SBP, systolic blood pressure.

## 5 EFFECT ON OBESITY

Obesity is an important global issue that is characterized by an imbalance between lipogenic and lipolytic processes, which causes the accumulation of excess body fat in the form of triglyceride in adipose tissue and is associated with several diseases including diabetes and heart disease (Langin, 2006).

As the approved anti‐obesity drugs have poorly documented effects, so, there is an immediate need for novel and effective anti‐obesity medicines (Kang & Park, 2012).

The research reported that ginseng exhibited an anti‐obesity effect by different mechanisms. Several studies have shown that ginseng in animals exhibited anti‐obesity effects.

Ginseng decreased adipose tissue mass and obesity in high‐fat diet‐induced obese mice and this effect mediated through the reduction of angiogenesis and extracellular matrix metalloproteinase (MMP) activity (Lee et al., 2013). In high‐fat diet‐induced obese mice, administration of 125 and 500 mg kg^−1^ day^−1^ of ginseng for 12 weeks decreased body and liver weight, epidermal adipose tissue weight through the downregulation of PPARγ expression, and upregulation of PPARα, PGC‐1α, UCP‐1, and UCP‐3 genes in adipose tissues (Chen et al., 2017).

The body weight‐lowering effect of ginseng extract (0.8% and 1.6% w/w) on obesity induced by a high‐fat diet in mice was investigated for 8 weeks. A significant decrease in plasma TG levels, body weight gains, and white adipose tissue were observed. The possible mechanism is through the regulation of lipogenesis‐related gene expression in white adipose tissue and delays in intestinal fat absorption (Lee et al., 2010). In a study on obese rats received a high‐fat diet, ginseng significantly reduced epididymal and abdominal adipose tissue mass and total body weight (Lee et al., 2017). In this regard, another experiment revealed that high hydrostatic pressure extract of ginseng (PEG) reduced the protein expression of adipogenic genes such as PPARγ and aP2. The results of this study showed PEG may have more useful than water extract ginseng on obesity and inflammation. This effect is mediated through the increase of fecal triacylglycerol and adjustment of gene expression (Jung et al., 2014). A study on the anti‐obesity effect of ginseng in 3T3‐L1 cells showed that ginsenoside Rg2 decreased adipocyte differentiation and the accumulation of intracellular lipids (Liu et al., 2019b). Ginseng decreased adipose tissue and adipocyte size, triglyceride, cholesterol, and body weight without changing food intake in high‐fat diet mice (Shin & Yoon, 2018).

Based on these studies, the anti‐obesity effect of ginseng and its active constituents is moderate to very strong. Furthermore, ginseng may have important roles in the treatment and prevention of obesity through several mechanisms (Table 3).

**TABLE 3:** Effects of ginseng on obesity

Study design	Constituents	Result	References
In vivo, Dog	GE, 1%, daily for 8 weeks	↓ BW, ↓ FM	Bae and Oh (2019)
In vivo, BALB/c mice, fed HFD	WEWG and WERG, 5.5 ml kg^−1^ day^−1^, for 10 weeks	Anti‐obesity effect of white ginseng was stronger than red ginseng	Zhou et al., (2018)
In vivo, mice, fed HFD	Mixture of FG (0.25%,0.5%,1%) and two probiotics, Bifidobacterium longum BORI and Lactobacillus paracasei CH88, daily for 9 weeks	↓ WG, ↓ lipid deposition in the liver and adipose tissues, ↓ Adipocyte size	Kang et al., (2018)
In vivo, Obese Sprague Dawley rats, fed HFD	GL, 3.3 mg kg^−1^ day^−1^	↓ BW, ↓ Epididymal and abdominal adipose tissue mass	Lee, Yoon, et al., (2017)
In vivo, Obese rats, Fed HFD	HPEG, 15 g/kg and WEG 15 g/kg for 14 weeks	PEG: ↓ BW and white adipose tissue mass Anti‐obesity effect of PEG was more than WEG	Jung et al., (2014)
In vivo, Mice, fed HFD	Cotreatment FG150 mg/kg/day and *Levan* 100 mg kg^−1^ day^−1^ for 11 weeks	↓ BW ↓ FM ↑ food efficiency ratio	Oh, Kwon, et al., (2014)
In vivo, Mice, fed HFD	HGV, 0.8 ml kg^−1^ day^−1^ for 8 weeks	↓ BW, ↓ fat weight, ↓ LW	Oh, Kwon, et al., (2014)
In vivo, Diet‐Induced Obese Mice	Ginsenoside Rb1, 20 mg kg^−1^ day^−1^, for 3 weeks	↓ WG ↓ FI	Lin et al., (2014)
In vivo, Mice, fed HFD	BGEE (1%,3%,5%) daily for 12 weeks	↓ FA in the liver, and white adipose tissues ↑ Total fecal weight ↑ Fecal fat excretion	Lee, Kim, et al., (2013)
In vivo, Obese‐prone C57BL/6J strain mice, Fed HFD	PG extracts, for 10 weeks	↓ WG	Woo et al., (2011)
In vivo, Mice, Fed HFD	Ginsenoside 0.02% and 0.05% (w/w),for 8 weeks	↓ Obesity ↓ Fatty liver	Liu et al., (2010)
In vivo, Obese insulin‐resistant rat	PG extract 300 mg kg^−1^ day^−1^ for 6 weeks	↓ BW ↓ LW ↓Serum alanine aminotransferase	Lim et al., (2009)
In vivo, obese mice, Fed HFD	Ginsenoside Rb2 40 mg kg^−1^ day^−1^ for 9 weeks	↓ BW	Gu et al., (2019)
In vivo, Obese C57BL/6 mice, fed HFD	FGR and FGB for 16 weeks	↓ WG ↓ FI FGR: ↓ Epididymal fat weight FGR : More potent anti‐obesity effect	Li, Li, et al., (2018)
In vivo Castrated C57BL/6J mice, fed HFD	GE, 5%, for 8 weeks	↓ BW ↓Adipose tissue mass ↓ Adipocyte size	Shin and Yoon (2018)
In vivo, C57BL/6J mice, fed HFD	Ginsenoside Rg1, 300 and 500 mg kg^−1^ day^−1^ for 8 weeks	↓ BW ↓ LA in white adipocyte tissue	Li et al., (2018)
In vivo, C57BL/6J mice, Fed HFD	Ginseng‐plus‐*Bai‐Hu‐Tang* 0.5% (w/w) for 12 weeks	↓ Obesity, ↓ Expansion of adipose tissue, ↓ Adipocyte hypertrophy	Lu et al., (2018)
In vivo, C57BL/6 mice, fed HFD	Saponins from PG, 125 and 500 mg kg^−1^ day^−1^ for 12 weeks	↓ BW ↓ LW ↓ Epididymal adipose tissue weight ↓ Food efficiency	Chen et al., (2017)
In vivo, C57BL/6J mice	Ginseng 5% (w/w) for 15 weeks	↓ BW ↓ Adipose tissue mass ↓ Adipocyte size	Lee et al., (2016)
In vivo, Rats, fed high‐fat/ high‐cholesterol diet	Cotreatment GBR and GE, 250 mg kg^−1^ day^−1^, for 12 weeks	↓ Obesity	Lee et al., (2015)
In vivo, Obese female db/db mice	GE, 4.5 g /kg/day, for 13 weeks	↓ BW ↓Adipose tissue mass ↓ Size of adipocytes in visceral adipose tissues	Lee et al., (2014)
In vivo, Mice, fed HFD	Ginseng, 0.5 g kg^−1^ day^−1^, for 15 weeks	↓ Body fat mass gain ↓ obesity	Li et al., (2014)
In vivo, Obese mice, Fed HFD	Cotreatment *Veratrum nigrum* 0.75g kg day^−1^ and PG 0.75g kg day^−1^, for 16 week	↓ fat weight, ↓ BW, ↓ LA	Park et al., (2013)
In vivo, Obese C57BL/6J mice, fed HFD	GE,0.5 and 5 (w/w) daily for 8 weeks	↓ Adipose tissue mass ↓ Obesity	Lee, Kim, et al., (2013)
In vivo, Mice, Fed HFD	Ginsenoside Rh2, 20 mg kg^−1^ day^−1^, for 4 weeks	↓ Adipogenesis, ↓ Adipocyte differentiation ↓ Body and epididymal fat weight gains	Gu et al., (2013)
In vivo, Obese mice, Fed HFD	GE, 0.8 and 1.6 (w/w) daily for 8 weeks	↓ White adipose tissue weight ↓ BW	Lee et al., (2010)
In vivo, Obese mice, Fed HFD	Ginsenoside Rg2, 10 mg kg^−1^ day^−1^, for 4 weeks	↓ BW	Liu et al., (2019b)
In vivo, Type 2 obese diabetic db/db mice	FSGB, 0.5 g kg^−1^ day^−1^, for 5 weeks	↓ BW	Kim et al., (2012)
In vivo, Otsuka Long‐Evans Tokushima fatty rats	PG, 200 mg/ kg/day for 40 weeks	↓ BW ↓ FM	Lee et al., (2009)
In vivo, Rat, Fed HFD	PG, 200 mg kg^−1^ day^−1^, for 18 weeks	↓ BW ↓ FM	Lee et al., (2012)
In vivo, Albino rat, Suffering in from acute liver diseases and diabetes	Ginseng (2% and 4%) and cotreatment (2% Ginseng and 0.25% Curcumin) and (4% Ginseng and 0. 5% Curcumin)	↓ BW ↓ FI ↓ Organs weight	Arafa (2013)
In vivo, Old‐aged, obese, leptin‐deficient mice(B6.V‐Lepob, “ob/ob”)	FRG, 0.5%, and 1.0% FRG for 16 weeks	↓ BW	Cheon et al., (2015)
In vivo, C57BL/6N mice, Fed HFD	Aged ginseng, 4.0%, for 8 weeks	↓ BW	Im Chung et al., (2016)
In vivo, STZ‐induced type 2 diabetic C57BL/6 mice, fed HFD	Ginsenoside Rk3, 10, 30, 60 mg kg^−1^ day^−1^, for 4 weeks	↓ LA	Liu et al., (2019c)
In vitro, 3T3‐L1 cells	WEGE	↓ LA	Park et al., (2015)
In vitro, 3T3‐L1 cells	Ginsenosides F2	↓ Adipogenesis	Siraj et al., (2015)
In vitro, 3T3‐L1 cells	Ginsenoside (95% purity)	↓ Lipid droplet accumulation, anti‐adipogenic effect	Simu et al., (2017)
In vitro, co‐culture system of 3T3‐L1 and RAW264.7 cells	Saponin fraction from ginseng 100 μg/mL	↓Obesity	Kim et al., (2018)
In vitro, 3T3‐L1 cells	Ginsenoside Rg2, 80 μM	↓ Adipocyte differentiation, ↓Accumulation of intracellular lipids	Liu et al., (2019b)
Human, Obese middle‐aged Korean women	Ginseng, 4 g/day for 8 weeks	↓ Body mass index ↓ BW	Song et al. (2014)

Abbreviations: BGEE, black ginseng ethanolic extract; BW, body weight; FBR, fermented black raspberry; FG, fermented ginseng; FGB, fermented ginseng berry; FGR, fermented ginseng root; FI, food intake; FM, fat mass; FRG, fermented Korean red ginseng; FSGB, fermented steam‐dried ginseng berries with *Lactobacillus plantarum;* GE, ginseng extract; GL, ginseng leaf; HFD, high‐fat diet; HGV, hydroponic‐cultured ginseng vinegar; HPEG, high hydrostatic pressure extract of ginseng; LA, lipid accumulation; LW, liver weight; PG, *Panax ginseng;* STZ, streptozotocin; WEG, hot water extract of ginseng; WEGE, water and ethanolic ginseng extracts; WERG, water extract of red ginseng; WEWG, water extract of white ginseng; WG, weight gain.

## 6 EFFECT ON HIGH BLOOD GLUCOSE

Diabetes is known as a metabolic disease that outcomes from failure in insulin action or insulin production or both (Mahadeva Rao & Adinew, 2011). Diabetes is one of the major reasons for human death, morbidity, and hospital cost around the world. According to the universal reports about diabetes, the number of people suffering from diabetes has been over 422 million in 2014 and the number of people with diabetes is increasing every day around the world (Collaboration, 2016). So, diabetes is a serious universal health problem, which is guessed to reach 592 million by 2035 and will be the seventh reason for mortality in 2035 (Das et al., 2014).

Many animal and human studies have shown useful effects of phytotherapy for the treatment of diabetes (Ghorbani, 2013a, 2013b).

Nowadays, the identification of suitable healthcare approaches, such as medicinal herbs, with fewer adverse effects is more appropriate, especially with attention to the undesirable side effects of chemical drugs. Avocado is a popular source of vitamins, minerals, carotenoids, phenolics, and fatty acids. The antidiabetic effects of avocado have been shown in several studies (Tab eshpour, Razavi, et al., 2017). *Nigella sativa* and its active component, thymoquinone, have been documented to show hypoglycemic properties (Razavi & Hosseinzadeh, 2014). Flavonoids such as rutin are useful in the treatment of many diseases such as diabetes (Hosseinzadeh & Nassiri‐Asl, 2014). The results of studies revealed that grape polyphenols reduce significantly the level of blood glucose (Akaberi & Hosseinzadeh, 2016).

Several mechanisms have been involved in the treatment of diabetes by phytochemicals. For example, reducing glucose absorption from the intestine, preventing glucose making in the liver, enhancing tissues glucose uptake, and increasing beta cell insulin secretion (Kamyab et al., 2010; Shafiee‐Nick et al., 2011, 2012).

Among different antidiabetic herbs, ginseng is one of the important accepted plants. The antidiabetic effects of ginseng and its active components have been described in numerous studies. For example, the extract of *P*. *ginseng* roots (120 mg/ kg) significantly decreased blood glucose level and improved glucose tolerance after 4 days of treatment in diabetic rats. These results suggested that ginseng extract has hypoglycemic effects on diabetic male rats (Liu et al., 2009).

The antidiabetic effect of ginseng was shown in a study on fatty mice, performed by Lee et al. This study demonstrated that ginseng upregulated the expression of genes involved in the activation of AMPK and increased mitochondrial biogenesis and glucose consumption in skeletal muscles (Lee et al., 2009). The ethanolic extract of ginseng significantly decreased the levels of fasting plasma glucose, HbAlc, and insulin resistance. On the other hand, the expression of phospho‐AMPK and glucose transporter 4 (GLUT4) were increased in liver and skeletal muscle in db/db mice (Do Yeon Kim et al., 2009). In another study, the oral administration of fermented red ginseng extract (100 and 200 mg/kg) for 3 weeks was able to significantly decrease the blood glucose level in streptozotocin‐diabetic rats (Kim et al., 2010). The daily administration of ginseng leaf extract (250 and 500 mg/kg) for 8 weeks in C57BL/6J mice, significantly reduced the plasma glucose level but increased the phosphorylation of AMP‐activated protein kinase (AMPK) and its substrate, acetyl‐CoA carboxylase. Moreover, phosphoenolpyruvate carboxykinase gene expression was reduced. These results suggest that ginseng leaf extract improved hyperglycemia by preventing gluconeogenesis and activating lipolysis, by AMPK stimulation (Yuan et al., 2010). In another study, the probable effect of ginseng on high blood glucose and related diseases was investigated. The findings of this study demonstrated that the modulatory effect on tumor necrosis factor‐alpha (TNF‐α) and interleukin‐6 (IL‐ 6) and liver antioxidants may be involved in the improvement of high blood glucose by ginseng in rats (El‐Khayat et al., 2011). Another study on the C2C12 skeletal muscle cells showed that ginseng (ginsenoside Rb1) increased glucose uptake and improved insulin sensitivity. The results showed that glucose uptake was mediated by leptin receptor activation. According to this study, the leptin receptor plays a great role in the ginseng effects on glucose uptake and insulin sensitivity in skeletal muscle cells (Tab andeh et al., 2017). A randomized double‐blind, placebo‐controlled, clinical trial study suggested that 8‐week supplementation with hydrolyzed ginseng extract (HGE) in 23 subjects demonstrated that fasting plasma glucose and postprandial glucose were significantly reduced in the HGE group in contrast to the placebo group (Park et al., 2014b). Randomized double‐blind cross over clinical trial on 24 subjects (F:M = 11:13; age = 64 ± 7 year; BMI = 27.8 ± 4.6 kg/m^2^; HbA1c = 7.1 ± 1.2%) for 8 weeks indicated that 3 g/day ginseng improved fasting blood glucose (−0.71 mmol/L; *p* = .008) and HbA1c (−0.29%; *p* = .041) (Vuksan et al., 2019).

In summary, ginseng can be suggested for the treatment of diabetic patients because it can decrease blood glucose levels with several effective mechanisms, such as insulin sensitivity improvement, the enhancement of tissues glucose uptake, and the reduction of insulin resistance and glucose tolerance. Numerous studies regarding the antidiabetic effect of the ginseng have been conducted on animals (mice and rats); thus, to demonstrate this effect on humans, we need more clinical research projects (Table 4).

**TABLE 4:** Effects of ginseng on high blood glucose

Study design	Constituents	Result	References
In vivo, STZ‐induced diabetic mice	Malonyl ginsenosides, 30, 60, 120 mg kg^−1^ day^−1^	↓ FBG Improved GT	Liu et al., (2009)
In vivo, Otsuka Long‐Evans Tokushima fatty rats	PG, 200 mg/ kg/day for 40 weeks	Improved IS preserved GT up to 50 weeks of age	Lee et al., (2009)
In vivo, C57BL mice	FG, 100, 200mg kg day^−1^ for 10 week	↓ FBG, ↓ HbAlc, ↓IR	Do Yeon Kim, Ahn, et al., (2009)
In vivo, Otsuka Long‐Evans Tokushima fatty rats	Ginsam, 300 and 500 mg kg^−1^ day^−1^ for 6 weeks	Improved GT ↓ fasting and postprandial glucose concentrations	Lim et al., (2009)
In vivo, Otsuka Long‐Evans Tokushima fatty rats	Ginsenoside, Rg3, 500 mg kg^−1^ day^−1^ for 8 weeks	Improved insulin signaling and GU	Kim et al., (2009b)
In vivo, STZ‐induced diabetic rats	BGE, 5 mg kg^−1^ day^−1^ for 3 weeks	↓ BG	Kim and Kang (2009)
In vivo, STZ‐induced diabetic rats	GR, 400 mg kg^−1^ day^−1^ for 6 weeks	↓ BG ↑ Preservation of β‐cells	Karaca et al., (2010)
In vivo, STZ‐induced diabetic rats	FRG, 100,200 mg kg^−1^ day^−1^ for 3 weeks	↓ BG, ↑ PIL, ↓ Activated of disaccharidases	Kim et al., (2010)
In vivo, C57BL/6J mice, Fed HFD‐induced hyperglycemia and hyperlipidemia	GLE, 250, 500 mg kg^−1^ day^−1^, for 8 weeks	↓ BG ↓Gluconeogenesis	Yuan et al., (2010)
In vivo, STZ‐induced diabetic rats	GR, 400 mg kg^−1^ day^−1^, for 6 weeks	↓ BG	Karaca et al., (2011)
In vivo, STZ‐induced diabetic rats	PG, 22.5 mg kg^−1^ day^−1^, for 45 days	↓ BG	El‐Khayat et al., (2011)
In vivo, Sprague Dawley rat, fed HFD	PG, 200 mg kg^−1^ day^−1^, for 18 weeks	↑ Insulin signaling ↑ IS	Lee, Lee, Lee, et al., (2012)
In vivo, STZ‐induced diabetic rats	PFRG, 300 mg kg^−1^ day^−1^, orally, for 5 weeks	↓ BG	Park, Kim, et al., (2012)
In vivo, C57BL/6J mice, fed HFD	Ginsenoside Re, 5, 10 and 20 mg kg^−1^ day^−1^ for 3 weeks	↓ BG	Quan et al., (2012)
In vivo, STZ‐induced diabetic rats	Ginsenoside Re, 40 mg kg^−1^ day^−1^ for 8 weeks	↓ BG	Liu et al., (2012)
In vivo, STZ‐induced diabetic mice	PGBE, 100 or 200 mg kg^−1^ day^−1^ for 10 weeks	↓ BG, Improved GT	Park, Kim, et al., (2012)
In vivo, ICR mice, fed HFD	PPGR,75, 150, 300 mg/Kg/day, orally, for 5 weeks	↓ Hyperglycemia	Yuan et al., (2012)
In vivo, Spontaneously diabetic GK rats	KRGWE, 0.2 g kg^−1^ day^−1^ for 12 weeks	↓ BG	Kim and Kim (2012)
In vivo, type 2 diabetic mice (db/db mice)	ARGME, 150 mg kg^−1^ day^−1^	↓ BG	Yoo et al., (2012)
In vivo, Wistar rat, fed HFD	Ginsenoside Re	↓ IR of skeletal muscle	Han et al., (2012)
In vivo, STZ‐induced diabetic C57BL/6J mice	KRGE, 25, 100, 1,000 mg kg^−1^ day^−1^, for 6 weeks	↓ BG	Hong, Kim, Lee, et al., (2012)
In vivo, STZ‐induced type 2 diabetic rat, fed HFD	PG, 50 and 100 mg kg^−1^ day^−1^, for 3 weeks	↓ FBG ↓ GTT ↓ IR	Liu et al., (2013)
In vivo, Diabetic C57BL mice	FGE, 0.1% (w/w), for 8 weeks	↓ BG ↓ HbAlc Improved GT	Jeon et al., (2013)
In vivo, Obese C57/L mice, fed HFD	Ginsenoside Rb1, 10 and 20 mg kg^−1^ day^−1^, for 2 weeks	Improved GT	Shang et al., (2013)
In vivo, Mice, Fed HFD	Combination of RGM (30%, w/w) and brown rice, for 8 weeks	Improved GM Hypoglycemic effect	Chung et al., (2014)
In vivo, Obese rat, fed HFD	Ginsenoside Rb1, for 5 days	Improved GT ↑ IS ↓ FBG	Shen et al., (2015)
In vivo, STZ‐induced diabetic rats	PRGP, 0.3%, 0.6%, for 6 weeks	↓ BG	Shim et al., (2015)
In vivo, C57BL/6 mice	GBE, 0.05%, for 24 or 32 weeks	↑ IS	Seo et al., (2015)
In vivo, Multiple low‐dose STZ‐induced diabetic rats	KGE, 100, 200, and 300 mg kg^−1^ day^−1^ for 8 weeks	Improved glucose homeostasis ↑ PIL	Moon et al., (2015)
In vivo, Old‐aged, obese, leptin‐deficient (B6.V‐Lepob, “ob/ob”) mice	FKRG, 0.5%, and FRG, 1.0% for 16 weeks	Improving IS ↓ BG	Cheon et al., (2015)
In vivo, Type 2 diabetic mice	Ginsenoside Rg3	↓ Hyperglycemic	Kim et al., (2015)
In vivo, STZ‐induced diabetic mice	BGE, 200 mg kg^−1^ day^−1^, for 5 weeks	↓ Hyperglycemia ↑ Insulin/glucose ratio	Kim, Pan, et al., (2016)
In vivo, C57BL/6N mice, fed HFD	Aged ginseng, 4.0%, for 8 weeks	Improved glucose metabolism ↓ BG	Im Chung et al., (2016)
In vivo, Type 2 diabetic rat	BGD, 37.2 g kg^−1^ day^−1^, for 2 weeks	↓ BG	Hou et al., (2017)
In vivo, Type 2 diabetic rats	Combination of freeze‐dried aronia, red ginseng, ultraviolet‐irradiated shiitake mushroom, and nattokinase, for 12 weeks	Improved GT ↓ IR	Yang et al., (2018)
In vivo, Type 2 diabetic mice	Aerobic exercise combined with panaxatriol, 0.2%, for 6 weeks	↓ IR	Takamura et al., (2017)
In vivo, Type 2 diabetic mice, fed HFD	Ginsenoside Rb1, 10 mg kg^−1^ day^−1^, for 1 week	↑ IS	Song et al., (2017)
In vivo, STZ‐induced diabetic mice	FRG with probiotics, 150 mg kg^−1^ day^−1^, for 8 weeks	↓ FBG	Jang et al., (2017)
In vivo, C57BL/6 Mice, Fed HFD	Ginsenoside Re, 5, 10 and 20 mg kg^−1^ day^−1^, for 4 weeks	↓ Hyperglycemia ↓ FBG ↓ IR	Kim et al., (2017a)
In vivo, Type 2 diabetic mice	GB, 0.05% or 0.1%, for 12 weeks	↓ BG ↓ Hyperglycemia ↓ IR	Kim et al., (2017b)
In vivo, Diabetes‐prone biobreeding rat	Diol‐GF, 1 mg g^−1^ day^−1^, for 8 weeks	Antidiabetogenic effect	Ju et al., (2019)
In vivo, STZ‐induced type 2 diabetic C57BL/6 mice, Fed HFD	Ginsenoside Rk3, 10, 30, 60 mg kg^−1^ day^−1^, for 4 weeks	↓ Hyperglycemia Improved GT ↓ IR	Liu et al., (2019b)
In vivo, Diabetic rat	GP and Ginsenoside Rb1, for 30 days	Synergistic antidiabetic effect	Li, Li, et al., (2018)
In vivo, High‐fat and high‐sugar induced hepatic insulin resistance in rat	Ginsenoside Rg1, 10, 25, 50 mg kg^−1^ day^−1^, for 4 weeks	↓ IR	Fan, Zhang, et al., (2019)
In vivo, C57BL/6J mice, Fed HFD	GLE, 250 and 500 mg kg^−1^ day^−1^, orally, for 8 weeks	↓ BG, ↓ Hyperglycemia	Yuan et al., (2010)
In vivo, Mice, Fed HFD	Cotreatment with FG, 150 mg kg^−1^ day^−1^ of with *Levan,* 100 mg kg^−1^ day^−1^ of for 11 weeks	↓ FBG ↓ IR	Oh, Kwon, et al., (2014)
In vivo, Diet‐Induced Obese mice	Ginsenoside Rb1, 20 mg kg^−1^ day^−1^, for 3 weeks	↓ BG	Lin et al., (2014)
In vivo, Type 2 obese diabetic db/db mice	FSGB with *Lactobacillus plantarum*, 0.5 g kg^−1^ day^−1^, for 5 weeks	↓ IR Improved GT	Kim et al., (2012)
In vivo, Obese insulin‐resistant rat	PGVE, 300 mg kg^−1^ day^−1^ for 6 weeks	↓ Fasting and postprandial glucose concentrations, Improved GT	Lim et al., (2009)
In vivo, Obese mice, Fed HFD	Ginsenoside Rb2 40 mg kg^−1^ day^−1^ for 9 weeks	Improved IS	Gu et al., (2019)
In vivo, Obese C57BL/6 mice, fed HFD	FGR 16 weeks	FGR: ↓ Hyperglycemia ↓ IR	Li, Li, et al., (2018)
In vivo, C57BL/6J mice, Fed HFD	Ginsenoside Rg1, 300 and 500 mg kg^−1^ day^−1^ for 8 weeks	↓ IR Improved GT	Li, Li, et al., (2018)
In vivo, C57BL/6J mice, Fed HFD	Ginseng‐plus‐*Bai‐Hu‐Tang* 0.5% (w/w) for 12 weeks	↑ IS Improved GT	Lu et al., (2018)
In vivo, C57BL/6 mice, Fed HFD	PG, 125 and 500 mg kg^−1^ day^−1^ for 12 weeks	↓ BG	Chen et al., (2017)
In vivo, ovariectomized C57BL/6J mice	Ginseng 5% (w/w) for 15 weeks	↓ IR ↓ Hyperglycemia	Lee, Bae, et al., (2016)
In vivo, obese C57BL/6J mice, fed HFD	GE, 0.5 and 5 (w/w) daily for 8 weeks	↑ IS Improved GT	Lee, Kim, et al., (2013)
In vitro, HepG2 cells	Ginsenoside Rg1	↑ GU ↓ IR ↓ Output of glucose	Fan, Tao, et al., (2019)
In vitro, C2C12 skeletal muscle cell	Ginsenoside Rb1, 0.1, 1, and 10 μM	↑ GU	Tab andeh et al., (2017)
In vitro, HepG2 cells	Ginsenoside Rb3, 25 µM	↓ Hepatic gluconeogenesis	Meng et al., (2017)
In vitro, C2C12 muscle cells	Ginsenoside Rg1	↑ GU ↓IR	Lee, Lee, Kim, et al., (2012)
In vitro, 3T3‐L1 cells	Ginsenoside Re	↓ IR	Gao et al., (2013)
In vitro, H4IIE cell line (rat hepatocytes)	Ginsenoside Rb2	↓ Abnormal hepatic gluconeogenesis on obesity‐induced	Lee et al., (2011)
In vitro, Mouse C2C12 muscle cells and INS−1 pancreatic β‐cells	Extracts from white, Taegeuk, and red ginseng root	TGRE: ↑ GU WGRE: ↑ Insulin‐induced glucose uptake	Cha et al., (2010)
Human, Type 2 diabetic subject	AG, 3 g/day, for 8 weeks	↓ FBG ↓ HbAlc	Vuksan et al., (2019)
Human, 23 Subject	HGE, 960 mg/day, for 8 weeks	↓ FBG ↓ Postprandial glucose	Park et al., (2014b)
Human, Lately diagnosed type 2 diabetic subject	KRG, 5 g/day, for 12 weeks	↓ BG	Bang et al., (2014)
Human, 45 Subjects	RGC, 20 g/day for 8 weeks	↓ FBG	Shin et al., (2011)
Human, 38 Subjects	FRG, 780 mg/day for 12 weeks	↓ FBG, ↓ HbAlc ↓IR	Kim et al., (2011)
Human, 93 postmenopausal women	FRG, for 2 weeks	↓ HbAlc ↓ IR ↓ Hyperglycemia	Lee et al., (2013)

Abbreviations: AG, American ginseng; ARGME, American Red Ginseng methanol extract; BG, blood glucose; BGD, *baihu ginseng* decoction; BGE, black ginseng extract; BGE, black ginseng extract; Diol‐GF, diol‐ginsenoside fraction from Korean red ginseng; FBG, fasting blood glucose; FG, fermented ginseng; FG, fermented ginseng; FGR, fermented ginseng root; FKRG, fermented Korean red ginseng; FRG, fermented red ginseng; FSGB, fermented steam‐dried ginseng berries; GB, ginseng berry; GBE, ginseng berry extract; GE, ginseng extract; GK, *Goto‐Kakizaki;* GLE, ginseng leaf extract; GLE, ginseng leaf extract; GM, glucose metabolism; GP, ginseng polysaccharides; GR, ginseng root; GT, glucose tolerance; GTT, glucose tolerance test; GU, glucose uptake; HFD, high‐fat diet; HGE, hydrolyzed ginseng extract; IR, insulin resistance; IS, insulin sensitivity; KGB, *Konjac*‐*glucomannan‐*based fiber blend; KGE, Korean ginseng extracts; KRG, Korean red ginseng; KRGE, Korean red ginseng extract; KRGWE, Korean red ginseng water extract; PG, *Panax ginseng;* PGBE*, Panax ginseng* berry extracts; PGVE, *Panax ginseng* vinegar extract; PIL, plasma insulin levels; PPGR, pectinase‐processed ginseng radix; PRGP, puffed red ginseng powder; RGC, red ginseng Cheonggukjang; RGM, red ginseng marc; STZ, streptozotocin; TGRE, *Taegeuk ginseng* root extracts; WGRE, white ginseng root extracts.

## 7 CONCLUSION

**FIGURE 2:**
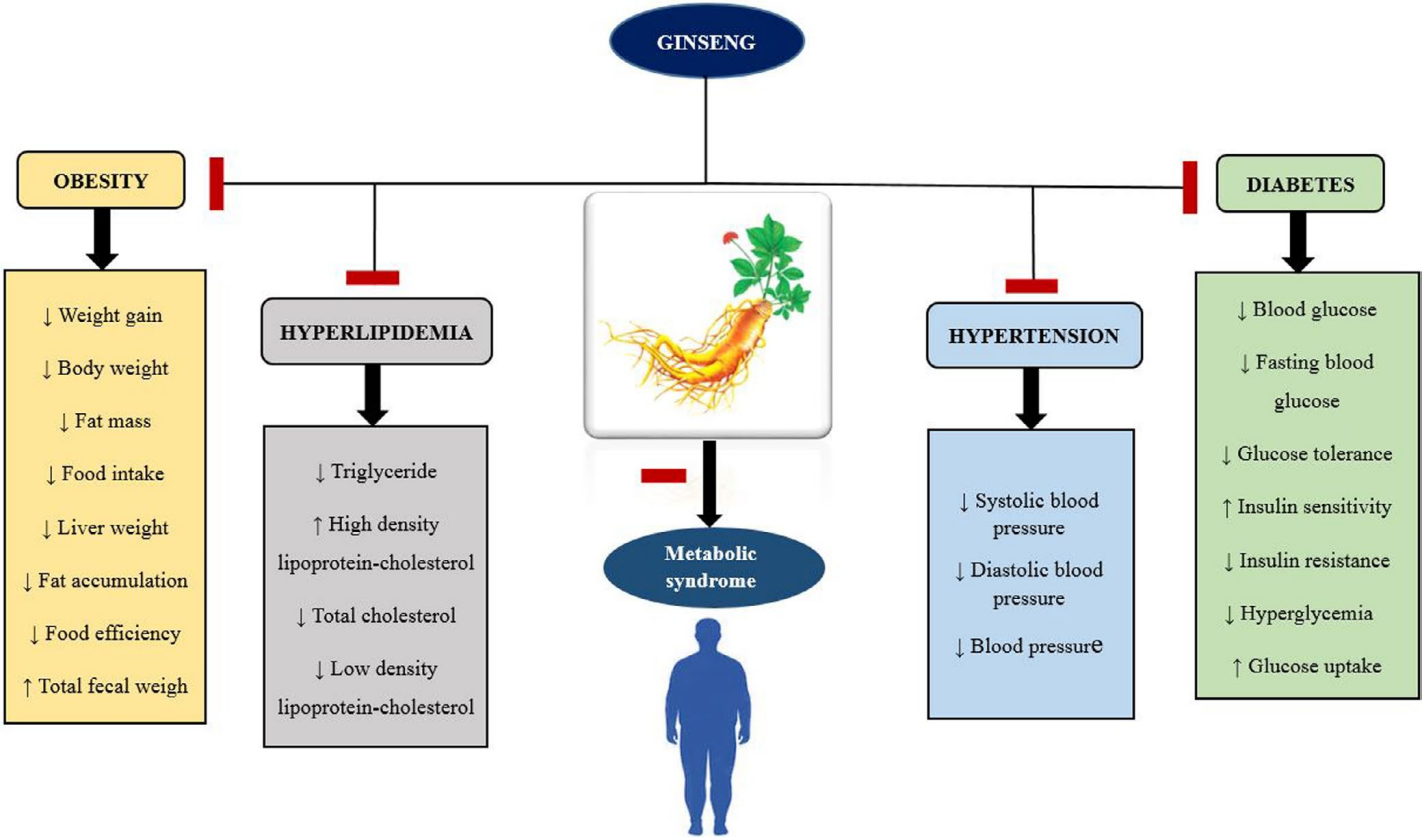
Schematic effects of ginseng in metabolic syndrome

The use of herbal medicines as supplementary drugs is prevalent and gaining global popularity. Ginseng has wide curative potentials including decreasing blood glucose level, blood lipids level, and blood pressure and enhancing insulin sensitivity. This review article summarizes a variety of in vitro, in vivo, and human studies on the role of ginseng and its active constituents in metabolic syndrome. The results of different studies have indicated that this plant exhibits useful effects in several components of metabolic syndrome including blood glucose, dyslipidemia, blood pressure, and obesity (Figure 2). Ginseng stimulates AMPK and activates lipolysis, so, it can improve hyperglycemia. Ginseng decreases adipose tissue mass and obesity. The possible mechanism is through the regulation of lipogenesis‐related gene expression. Treatment by ginseng can improve hyperlipidemia and stimulate lipolysis by AMPK activation. Ginseng is useful in decreasing high blood pressure through eNOS activation and enhances NO‐releasing. As ginseng does not induce important side effects, so it can be used as an herbal medicine for the treatment of various components of metabolic syndrome. However, more clinical studies need to be done for confirming the beneficial effects of ginseng in metabolic syndrome.

## CONFLICT OF INTEREST STATEMENT

The authors declare that there are no conflicts of interest.

## AUTHOR CONTRIBUTION

**Tahereh Aminifard :** Data curation (equal); Formal analysis (equal); Methodology (equal); Writing‐original draft (equal). **Marjan Razavi:** Conceptualization (equal); Data curation (equal); Formal analysis (equal); Supervision (equal); Writing‐review & editing (equal). **Hossein Hosseinzadeh:** Conceptualization (equal); Data curation (equal); Formal analysis (equal); Investigation (equal); Supervision (equal); Validation (equal); Writing‐review & editing (equal).
